# Comparative study of the effects of gold and silver nanoparticles on the metabolism of human dermal fibroblasts

**DOI:** 10.1093/rb/rbz051

**Published:** 2020-01-25

**Authors:** Yan Huang, Xiaoying Lü, Rong Chen, Ye Chen

**Affiliations:** State Key Laboratory of Bioelectronics, School of Biological Science and Medical Engineering, Southeast University, Nanjing 210096, P.R. China

**Keywords:** gold and silver nanoparticles, human dermal fibroblasts, metabolomics and bioinformatics, metabolic pathway

## Abstract

The purpose of this article was to explore the effects of gold nanoparticles (GNPs) and silver nanoparticles (SNPs) with different cytotoxicities on human dermal fibroblasts (HDFs) at the metabolic level. First, ∼20 nm of GNPs and SNPs were prepared, and their effects on the proliferation of HDFs were evaluated. Then, a metabolomics technique was used to analyse the effects of GNPs and SNPs on the expression profiles of metabolites in HDFs after 4, 8 and 24 h of treatment. Furthermore, the key metabolites and key metabolic pathways involved in the interaction of GNPs and SNPs with HDFs were identified through expression pattern analysis and metabolic pathway analysis of differentially expressed metabolites and were finally verified by experiments. The results of the cytotoxicity experiments showed that there was no cytotoxicity after the treatment of GNPs for 72 h, while the cytotoxicity of the SNPs reached grade 1 after 72 h. By using metabolomics analysis, 29, 30 and 27 metabolites were shown to be differentially expressed in HDFs after GNP treatment, while SNPs induced the differential expression of 13, 33 and 22 metabolites after 4, 8 and 24 h of treatment, respectively. Six and four candidate key metabolites in the GNP and SNP groups were identified by expression pattern analysis and metabolic pathway analysis, respectively. The key metabolic pathways in the GNP and SNP groups were identified as the glutathione metabolic pathway (the key metabolite of which was glutathione) and the citrate cycle pathway (the key metabolite of which was malic acid). Based on the experiments used to verify the key metabolites and key metabolic pathways, it was found that the increase in glutathione after GNP treatment might trigger an oxidative stress protection mechanism and thus avoid cytotoxicity. After exposure to SNPs, the citric acid content was increased, mainly through the citrate cycle pathway, thereby inhibiting the synthesis of malic acid to affect the formation of ATP and finally leading to cytotoxicity.

## Introduction

The use of functional metal-based nanoparticles has increased exponentially over the last decade. The biosafety and toxicity of nanoparticles have been widely studied by researchers. Although data on the toxicity of different nanomaterials in different cell lines and animal models have been obtained through recent studies, the molecular mechanisms and factors influencing nanomaterial toxicity are still poorly understood [1]. Therefore, ‘the mechanism by which nanomaterials act at different levels of biological systems, including molecules, cells, tissues and biological organs’ has become an extremely important topic in nanotoxicology research [2].

As many activities in cells occur at the metabolite level, intercellular signal transduction, energy transfer, and cell proliferation and differentiation are all regulated by metabolites, and metabolic changes are the end result of the expression of the functional genome, transcriptome and proteome [3]; thus, the analysis of metabolites has become important in the study of the molecular basis of life activities. Researchers have found that nanoparticles could affect the expression of intracellular metabolites [3–8].

Traditional methods for studying changes in metabolite expression include enzyme-linked immunosorbent assays, isotope labelling and fluorescent labelling. These methods can only measure known, single or small amounts of metabolites. In fact, external stimuli can induce an overall change in endogenous metabolites and have a ‘cascade’ effect on metabolic pathways. Therefore, traditional methods cannot be used to explore the changes of the metabolic system as a whole and thus cannot explain the mechanism of action at the metabolic level. High-throughput metabolomics technology based on nuclear magnetic resonance, LC/MS and GC-MS can determine the complete metabolite expression profile of cells. Therefore, metabolomics has become a new method to study the cytotoxicity mechanism of nanoparticles and plays an important role in revealing the unpredictable biological effects of nanoparticles and discovering new endpoint markers.

Gold nanoparticles (GNPs) and silver nanoparticles (SNPs) are two kinds of nanomaterials commonly used in biomedical applications. GNPs are used as gene and drug carriers and for medical imaging, rheumatoid arthritis treatment and tumour treatment [9]. GNPs can also be fabricated to a nanoarray with RGD grafted on it on inorganic and polymeric substrates to reveal the science of interactions between cells and biomaterials [10–12]. As a new inorganic antibacterial material with high stability and antidrug resistance, SNPs are used as medical antibacterial agents and antibacterial coatings. Some studies have shown that both GNPs and SNPs could affect the metabolic function of cells [13–15]. Mironava *et al.* found that 13 and 45 nm GNPs could reduce lipid accumulation in human adipose-derived stromal cells after 1 week by using Oil Red O staining and confocal microscopy [9]. However, no reports on the expression of metabolites in cells affected by GNPs measured by metabolomics technologies have been published. Lee *et al.* investigated the effects of 5 and 100 nm SNPs on glucose metabolism in HepG2, Huh7 and THP-1 cells with a biochemical analyser. The results showed that 5 nm SNPs reduced the release of lactic acid in HepG2 and Huh7 cells and decreased the consumption of glucose in HepG2 cells [15]. Carrola *et al.* analysed the effect of 30 nm SNPs on the expression profile of metabolites in human epidermal keratinocytes by using NMR-based metabolomics techniques, and 23 differentially expressed metabolites were identified [1]. However, in-depth analysis of the involved metabolic pathways and toxicity mechanisms have not been conducted. To date, studies comparing the effects of GNPs and SNPs on cells at the metabolic level and exploring key metabolic pathways have not been reported.

The purpose of this article is to compare the effects of GNPs and SNPs on human dermal fibroblasts (HDFs) at the metabolic level. First, the effects of these two nanoparticles on cell proliferation at the cellular level were compared. Then, metabolomics technologies were used to screen the differentially expressed metabolites affected by GNPs and SNPs, and metabolic pathway analysis was carried out to identify the key metabolites and key pathways involved in the interactions of the two nanoparticles with HDFs. Afterwards, the functions of key metabolic pathways were verified through verification experiments, and finally, the molecular mechanism underlying the differences in the cytotoxicity of GNPs and SNPs was elucidated.

## Materials and methods

### Preparation and characterization of GNPs and SNPs

GNPs ∼20 nm in size were prepared by the sodium citrate reduction of chloroauric acid [16], and SNPs ∼20 nm in size were prepared by the sodium borohydride reduction of silver nitrate [17]. The morphology of GNPs and SNPs was observed with transmission electron microscopy (TEM) (JEOL JEM-2100, Japan), and their sizes were determined with Image-Pro Plus software v6.0 (Media Cybernetics, Inc., USA). The concentrations of the GNPs and SNPs were measured using an inductive coupled plasma-optical emission spectrometer (Optima 5300DV, Perkin Elmer, USA).

### Cell culture

The HDFs were a gift from the Institute of Dermatology, Chinese Academy of Medical Sciences (Jiangsu, China). HDFs were cultured in low-glucose DMEM (HyClone, USA) supplemented with 10% foetal bovine serum (Biological Industries, Israel) and 1% (v/v) penicillin and streptomycin solution (Biological Industries, Israel) at 37°C in 5% CO_2_. After reaching 80–90% confluence, the HDFs were collected for subsequent experiments.

### Evaluation of the cytotoxicity of GNPs and SNPs towards HDFs

A total of 100 μl of HDFs with a concentration of 6 × 10^4^ cells/ml was added to a 96-well plate. After 24 h, the culture medium was aspirated, and 200 μM GNPs/SNPs (20 nm) were added, after which the HDFs were treated for another 4, 8, 24 or 72 h. The cytotoxicity of the GNPs/SNPs was evaluated by the MTT method [18]. Cells cultured in medium without GNPs or SNPs were used as the negative control, and cells cultured in medium containing 0.7% acrylamide were used as the positive control.

### Metabolomics analysis of the interactions between GNPs, SNPs and HDFs

#### Metabolomics experiment

Five millilitres of HDFs with a concentration of 2 × 10^6^ cells/ml were cultured in tissue culture polystyrene bottles (Corning, USA) with a bottom area of 25 cm^2^. After 24 h, the culture medium was aspirated, and 3 ml of 200 μM GNPs/SNPs was added. Cell samples were collected after 4, 8, and 24 h of treatment, with untreated HDFs were used as a control. Then, the total intracellular metabolites were extracted with precooled methanol. The separation and identification of the metabolites were performed with an Agilent 1290 Infinity LC system coupled to an Agilent 6530 Accurate Mass Q-TOF/MS. The metabolite expression profiles were obtained in both positive and negative ion mode (Shanghai Sensichip Hightech, Shanghai, China). The experiments were repeated five times for each group.

#### Screening of differentially expressed metabolites and important differentially expressed metabolites

After pre-processing, post-editing and normalization of the LC/MS data, unsupervised principal component analysis (PCA) was conducted with Simca-P (version 13.0) to evaluate the differences within and between the groups. The differentially expressed metabolites were then determined according to the variable importance in projection value (threshold > 1) obtained from the orthogonal partial least squares-discriminant analysis (OPLS-DA) and the *P* values obtained with Student’s *t*-test (*P *<* *0.05). The fold changes of the metabolites were calculated (using base 2 to calculate the log ratio of the experimental group/control group), and the significantly up- (fold change of >0) or downregulated (fold change of <0) metabolites were identified. The differentially expressed metabolites were further determined by comparing the mass-to-charge ratio (*m*/*z*) or the molecular mass and searching the online database (http://metlin.scripps.edu/). Finally, metabolites that were differentially expressed at least two different time points with the same expression patterns were identified as important differentially expressed metabolites.

### Screening of the candidate key metabolites affected by GNPs and SNPs

First, the MetaboAnalyst (https://www.metaboanalyst.ca/) online tool was used to analyse the metabolic pathways affected by the important differentially expressed metabolites influenced by the GNPs and SNPs that were identified in Section Screening of differentially expressed metabolites and important differentially expressed metabolites. Then, the candidate key metabolites involved in the interactions between GNPs/SNPs and HDFs were determined according to the following criteria: (i) identified as an important differentially expressed metabolite and (ii) involved in at least one metabolic pathway.

### Screening of the key metabolic pathways affected by GNPs and SNPs

A metabolic pathway analysis was performed with MetaboAnalyst for the metabolites differentially expressed in HDFs after treatment with GNPs/SNPs for 4, 8 and 24 h. The key metabolic pathways involved in the interactions between GNPs/SNPs and HDFs were determined according to the following criteria: (i) affected by differentially expressed metabolites at all three time points, (ii) contained the candidate key metabolites and (iii) had the greatest pathway impact. The key metabolites were further identified by molecular functional analysis of candidate key metabolites involved in key metabolic pathways.

### Verification of the expression levels of key metabolites involved in key metabolic pathways and the pathway functions affected by GNPs and SNPs

#### Verification of the expression levels of key metabolites

The expression level of glutathione in the GNP group was analysed by an LC-MS platform (UHPLC-LTQ/MS, Thermo Scientific, USA). The expression level of malic acid in the SNP group was analysed by using a GC-MS platform (7890B-5977A, Agilent, USA). The experiments were carried out by Shanghai Wiki Biotechnology Co., Ltd.

#### Verification of the functions of key metabolic pathways

##### Determination of the total glutathione content

Five millilitres of HDFs were cultured in tissue culture polystyrene bottles with a bottom area of 25 cm^2^. Once the cells had grown to 90% confluence, the culture media were replaced with 2.7 ml of 200 μM GNPs. After culturing for another 4, 8 or 24 h, the cells were collected by trypsinization, and the samples were prepared according to the instructions of the GSH and GSSG test kits (S0053, Shanghai Biyuntian Biotechnology Co., Ltd., China). The total glutathione content in each sample was determined by a microplate reader (Multiskan GO, Thermo Fisher Scientific, USA). HDFs cultured in culture medium without GNPs were used as controls.

##### Measurement of ATP content

The intracellular ATP contents in SNP-treated HDFs were measured with an ATP Analysis Kit (Beyotime, China). A total of 1 ml of HDFs with a concentration of 6 × 10^4^/ml was cultured in a 12-well plate. After 24 h, the culture solution was aspirated, and 1 ml of 200 μM SNPs was added. After 4, 8 and 24 h of treatment, the HDFs were lysed, and the supernatants were collected. The ATP concentrations (*C*_ATP_) and protein concentrations (*C*_Protein_) were then detected, and the ATP content was determined as *C*_ATP_/*C*_Protein_ and expressed as nmol/mg [17]. HDFs cultured in culture medium without SNPs were used as controls.

##### Detection of citrate content

Five millilitres of HDFs were cultured in tissue culture polystyrene bottles with a bottom area of 25 cm^2^. After the cells had reached 90% confluence, the culture media were aspirated, and 2.7 ml of 200 μM SNPs was added. After treatment with SNPs for another 4, 8 or 24 h, the cells were collected by trypsinization, and the samples were prepared according to the instructions of the Citrate Assay Kit (MAK057, Sigma, USA). The citrate content in each sample was measured with a microplate reader. HDFs cultured in culture medium without SNPs were used as controls.

### Statistical analysis

All experimental data were expressed as the mean ± standard deviation (SD). Student’s *t*-test was performed unless otherwise noted. *P *<* *0.05 was considered to indicate significant difference, and *P *<* *0.01 was considered to indicate a very significant difference. All experiments were repeated at least three times.

## Results and discussion

### Preparation and characterization of GNPs and SNPs

**Figure 1:**
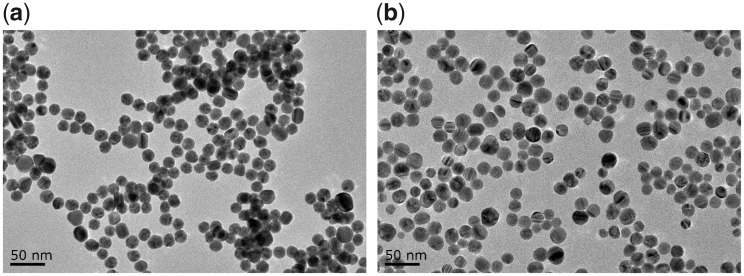
TEM Images of (**a**) GNPs and (**b**) SNPs

Figure 1 shows TEM images of GNPs and SNPs. Both types of nanoparticles were spherical and monodispersed, with average diameters and SDs of 20.7 ± 2.5 and 20.8 ± 2.4 nm, respectively.

### Evaluation of the cytotoxicity of GNPs and SNPs towards HDFs

**Figure 2:**
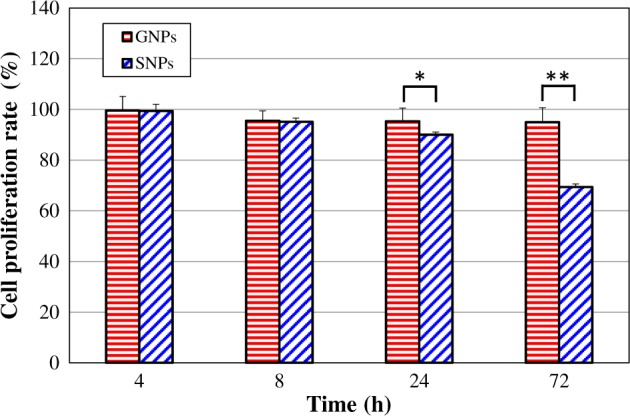
Cell proliferation rates of HDFs treated with GNPs/SNPs for 4, 8, 24 and 72 h. The results are presented as the mean ± SD (*n* = 6). *P <* *0.05, **P <* *0.01. A cytotoxicity grade of 0 indicates *P* ∈ [81%, 100%], grade 1 indicates *P* ∈ [61%, 80%], grade 2 indicates *P* ∈ [41%, 60%], grade 3 indicates *P* ∈ [21%, 40%] and grade 4 indicates *P* ∈ [0, 20%]

The cell proliferation rates of HDFs treated with 20 nm GNPs/SNPs for 4, 8, 24 and 72 h are shown in Fig. 2. All the cell proliferation rates for GNPs were higher than 90%, and the GNPs showed no cytotoxicity. For SNPs, the proliferation rate decreased to 69.3% after 72 h, and the cytotoxicity grade was 1. In addition, although the cytotoxicity grade of both the GNPs and SNPs was 0 at 24 h, the proliferation rate of SNP-treated HDFs was significantly lower than that of GNP-treated HDFs (*P *<* *0.05). At 72 h, the cell proliferation rate of the SNP group was very significantly lower than that of the GNP group (*P *<* *0.01). The results indicated that the cytotoxicity of SNPs was greater than that of GNPs.

Comfort *et al.* found that 10 nm GNPs with concentrations of 5, 25 and 100 μg/ml were non-toxic towards A-431 cells, and the cell viability remained unaltered after 24 h. However, 10 nm SNPs induced a strong cytotoxic response at concentrations ≥25 µg/ml (with a cell proliferation rate < 60%) [19]. Aueviriyavit *et al.* used MTT and trypan blue exclusion assays to investigate the cytotoxic effects of SNPs and GNPs (<100 nm, concentration range 5–1000 μg/ml) on the Caco-2 cell line after 24 h of exposure. A dose-dependent toxic effect of SNPs on Caco-2 cells was found, with estimated IC50 values of 16.7 μg/ml (MTT assay) and 14.9 μg/ml (trypan blue exclusion assay), respectively. GNPs did not cause a significant decrease in cell viability, even at a concentration of up to 1000 μg/ml [20]. Parveen *et al.* determined the effect of 21 nm GNPs and 20 nm SNPs on human carcinoma cell lines (A549, LNCap-FGC and MDA-MB) using an MTT assay. The IC50 for SNPs was lower than that for GNPs, indicating the increased cytotoxicity of SNPs [21]. The results obtained in this study showed that the cytotoxicity of SNPs was greater than that of GNPs, which was consistent with the results of the above studies [19–21].

### Metabolomics analysis of the interactions between GNPs, SNPs and HDFs

#### Screening of differentially expressed metabolites

The metabolite expression profiles in untreated and GNP- and SNP-treated HDFs were determined by LC/MS metabolomics analysis. By using PCA analysis, OPLS-DA analysis and *t*-tests, the differentially expressed metabolites were screened, and the results are summarized in Table 1. Detailed information about the differentially expressed metabolites is listed in Supplementary Tables S1–S6. There were 29, 30 and 27 differentially expressed metabolites in the GNP-4h, GNP-8h and GNP-24h groups, and 13, 33, and 22 differentially expressed metabolites in the SNP-4h, SNP-8h and SNP-24h groups, respectively.

**Table 1:** Quantitative analysis of the differentially expressed metabolites in HDFs treated with GNPs and SNPs for 4, 8 and 24 h

Group	GNPs			SNPs		
	4 h	8 h	24 h	4 h	8 h	24 h
Upregulated metabolites	16	13	18	6	24	5
Downregulated metabolites	13	17	9	7	9	17
Differentially expressed metabolites	29	30	27	13	33	22
Total of the differentially expressed metabolites at all three time points	58			52		

#### Screening of important differentially expressed metabolites

The differentially expressed metabolites (Table 1 and Supplementary Tables S1–S6) were analysed, and the important differentially expressed metabolites that were differentially expressed at least two time points with the same expression pattern were screened and are shown in Tables 2 and 3. It could be observed that there were 15 and 8 important differentially expressed metabolites in the GNP group (Table 2) and SNP group (Table 3), respectively. Among them, only two metabolites (glutathione and oxododecanoic acid) were common to both the GNP and SNP groups, and glutathione exhibited an opposing expression pattern (upregulated in the GNP group and downregulated in the SNP group), while oxododecanoic acid exhibited the same expression pattern in both groups (downregulated). Overall, there were significant differences in the important differentially expressed metabolites affected by the two types of nanoparticles, suggesting that the difference in cytotoxicity caused by the two types of nanoparticles might be related to their differing effects on the metabolite expression profile. By comparing the important differentially expressed metabolites identified in this article (Tables 2 and 3) with the nanoparticle-induced differentially expressed metabolites identified by other researchers, it was found that four metabolites (glutathione, uridine, malic acid and xanthine) had been reported to be affected by SNPs, TiO_2_ and CoFe_2_O_4_ nanoparticles [1, 4, 5, 7, 8, 22], and the other 17 metabolites were identified only in this study.

**Table 2:** Important differentially expressed metabolites and their fold change values at three time points in the GNP group

No.	Important differentially expressed metabolites	GNPs-4h	GNPs-8h	GNPs-24h
1	Glutathione	3.294	3.451	2.839
2	PE (20:4)	0.580	0.322	0.923
3	PE (22:4)	0.547	0.618	0.869
4	PE (22:6)	0.512	0.445	1.961
5	Linoleamide	1.948	2.134	
6	Leukotriene C4	1.505	1.386	
7	Arachidonic acid	0.701	0.536	
8	Docosahexaenoic acid	0.482	0.379	
9	PC (13:0)/PE(16:0)	0.284		0.839
10	Uridine	−0.260	−0.262	
11	Oxododecanoic acid	−0.344		−0.196
12	Indolelactic acid	−0.361	−0.413	
13	Chenodeoxycholic acid glycine conjugate	−0.499	−0.429	
14	DiHODE/HpODE	−0.513	−0.639	
15	Decanoyl-l-carnitine	−1.286	−2.025	

**Table 3:** Important differentially expressed metabolites and their fold change values at three time points in the SNP group

No.	Important differentially expressed metabolites	SNPs-4h	SNPs-8h	SNPs-24h
1	Malic acid	−1.277	−0.923	−19.181
2	Hydroxyvaleric acid	2.894	2.916	
3	Xanthine	2.349	1.815	
4	Cytosine		16.078	14.383
5	Oxododecanoic acid	−0.339	−1.111	
6	Anandamide (20:2, *n*-6)	−1.439		−2.645
7	Glutathione	−3.560	−2.925	
8	PC(19:3)		−1.757	−19.755

### Screening of candidate key metabolites affected by GNPs and SNPs

Some metabolites in the metabolite expression profile are outside the metabolic pathways, while those important metabolites are involved in specific metabolic pathways. These important metabolites are produced by the functional unit in metabolic networks, i.e. the actual enzyme or gene product via executing a particular chemical reaction or facilitating a transport process [23]. Therefore, studies of metabolite expression profiles often focus on the analysis of these important metabolites that participate in specific metabolic pathways. Compared to the metabolites that are not involved in any metabolic pathway, the analysis of important metabolites that involved in specific metabolic pathways is meaningful for understanding the transcriptional regulation from enzymatic activation/inhibition to genetic level after the action of materials on cells [23], and then explaining the interaction mechanism between materials and cells. So only the important differentially expressed metabolite that involved in at least one metabolic pathway was defined as candidate key metabolite in this article.

The metabolic pathways affected by the important differentially expressed metabolites in the GNP and SNP groups (Tables 2 and 3) were analysed with MetaboAnalyst and are listed in Tables 4 and 5. There were six important differentially expressed metabolites in the GNP group that were involved in six metabolic pathways (Table 4), and there were four metabolites in the SNP group that were involved in eight metabolic pathways (Table 5). Based on the above discussion and metabolic pathway analysis results of important differentially expressed metabolites, the six important differentially expressed metabolites listed in Table 4 and the four metabolites in Table 5 compliant with the screening criteria for candidate key metabolite, so they could be confirmed as the candidate key metabolites in the GNP and SNP group, respectively.

**Table 4:** Metabolic pathways affected by the important differentially expressed metabolites in the GNP group

No.	Important differentially expressed metabolites	Metabolic pathway
1	Glutathione	Glutathione metabolism pathway Cysteine and methionine metabolism pathway
2	Leukotriene C4	Arachidonic acid metabolism pathway
3	Arachidonic acid	Arachidonic acid metabolism pathway
4	Uridine	Pyrimidine metabolism pathway
5	Indolelactic acid	Tryptophan metabolism pathway
6	Chenodeoxycholic acid glycine conjugate	Primary bile acid biosynthesis pathway

**Table 5:** Metabolic pathways affected by the important differentially expressed metabolites in the SNP group

No.	Important differentially expressed metabolites	Metabolic pathway
1	Malic acid	Citrate cycle pathway Glyoxylate and dicarboxylate metabolism pathway Pyruvate metabolism pathway
2	Xanthine	Purine metabolism pathway Caffeine metabolism pathway
3	Cytosine	Pyrimidine metabolism pathway
4	Glutathione	Glutathione metabolism pathway Cysteine and methionine metabolism pathway

### Screening of the key metabolic pathways affected by GNPs and SNPs

According to the first screening criterion for the key metabolic pathways described in Section Screening of the key metabolic pathways affected by GNPs and SNPs in Materials and methods (pathway was affected by differentially expressed metabolites at all three time points), the metabolic pathway analysis of the differentially expressed metabolites (Table 1 and Supplementary Tables S1–S6) at each time point in the GNP and SNP groups was performed, and the details are listed in Supplementary Tables S7–S12. Six metabolic pathways were found to be affected by the differentially expressed metabolites in HDFs after GNP treatment for 4, 8 and 24 h (Table 6), and three metabolic pathways were found to be affected in the SNP group (Table 7).

**Table 6:** Metabolic pathways affected after HDFs were treated with GNPs for 4, 8 and 24 h and the involved metabolites and pathway impact

No.	Metabolic pathway	GNPs-4h		GNPs-8h		GNPs-24h	
		Metabolite	Pathway impact	Metabolite	Pathway impact	Metabolite	Pathway impact
1	d-Glutamine and d-Glutamate metabolism pathway	Glutamate	0.326	Glutamate	0.326	Glutamate	0.326
2	Glutathione metabolism pathway	Glutathione	0.237	Glutathione Pyroglutamic acid	0.239	Glutathione	0.237
3	Pyrimidine metabolism pathway	Uridine	0.021	Uridine	0.021	Cytosine	0.021
4	Vitamin B6 metabolism pathway	Glutamate	0.008	Glutamate	0.008	Glutamate	0.008
5	Cysteine and methionine metabolism pathway	Glutathione	0.007	Glutathione	0.007	Glutathione	0.007
6	Sphingolipid metabolism pathway	Phytosphingosine	0	Sphingosine Phytosphingosine	0.091	Sphinganine Phytosphingosine Sphingosine	0.231

**Table 7:** Metabolic pathways affected after HDFs were treated with SNPs for 4, 8 and 24 h and the involved metabolites and pathway impact

No.	Metabolic pathway	SNPs-4h		SNPs-8h		SNPs-24h	
		Metabolite	Pathway impact	Metabolite	Pathway impact	Metabolite	Pathway impact
1	Citrate cycle pathway	Malic acid	0.044	Malic acid	0.044	Malic acid	0.044
2	Glyoxylate and dicarboxylate metabolism pathway	Malic acid	0.024	Malic acid	0.024	Malic acid	0.024
3	Pyruvate metabolism pathway	Malic acid	0	Malic acid	0	Malic acid	0

**Figure 3:**
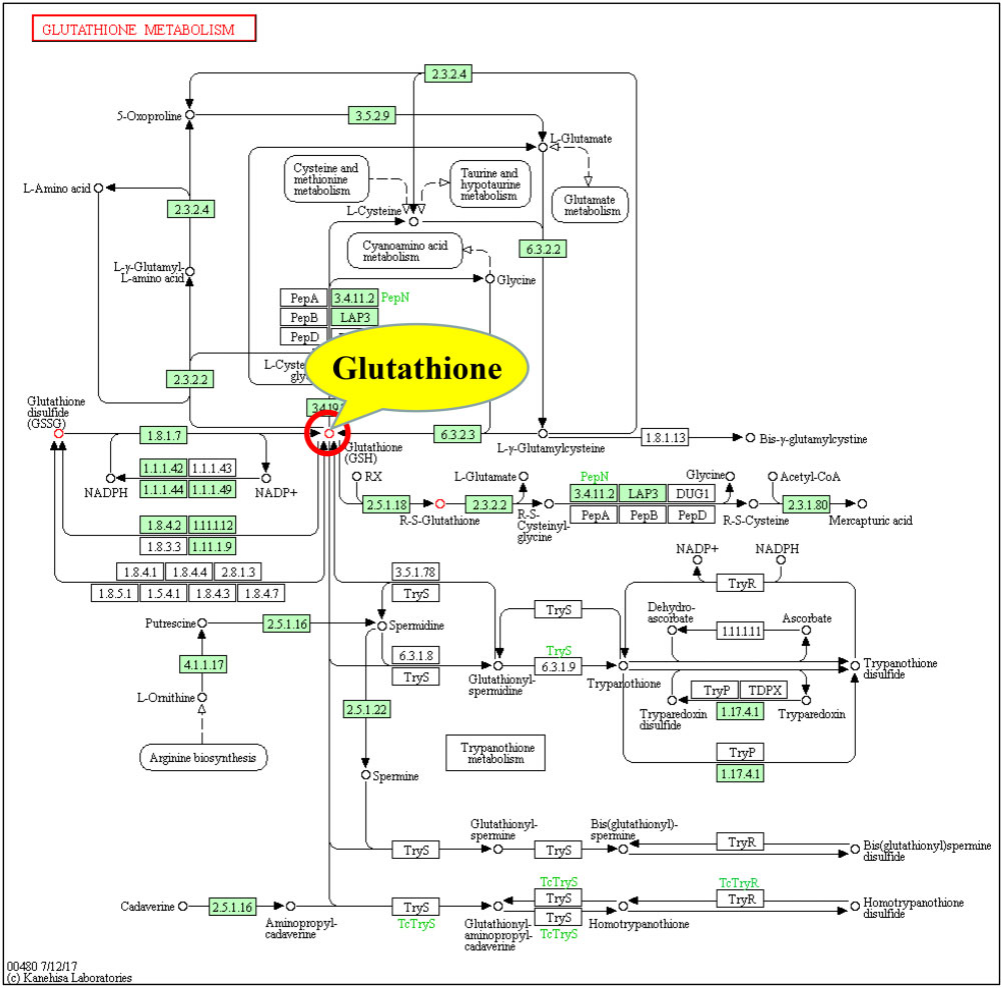
Key metabolic pathways (glutathione metabolic pathway) [24] involved in the interaction between GNPs and HDFs and the key metabolites involved

**Figure 4:**
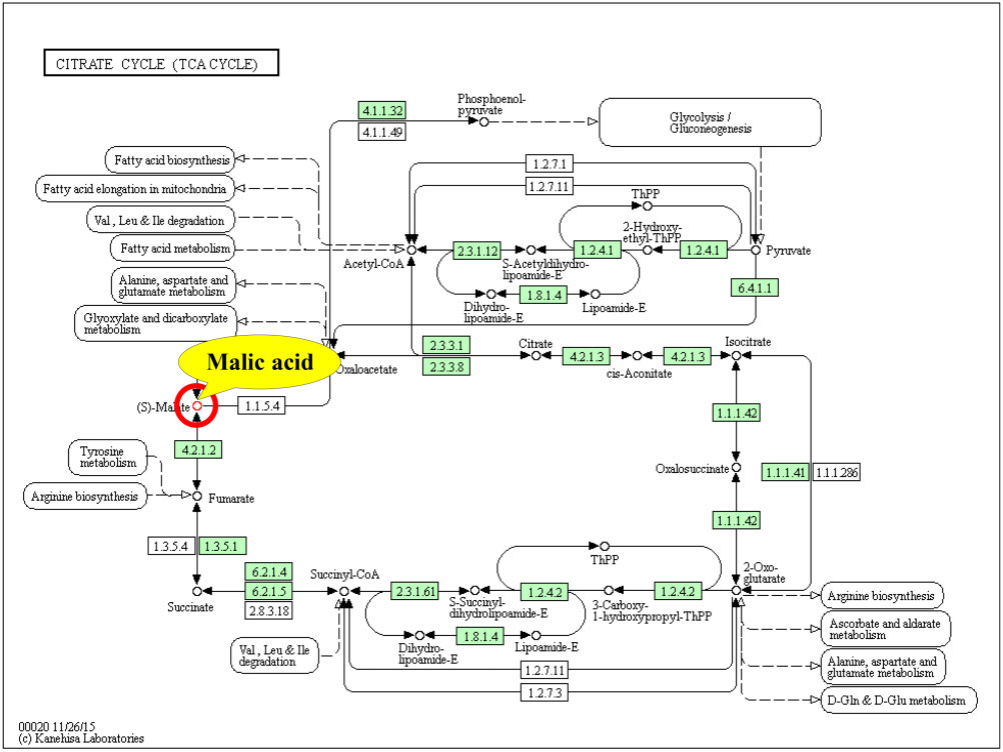
Key metabolic pathways (citrate cycle pathway) [24] involved in the interaction between SNPs and HDFs and the key metabolites involved

Furthermore, according to the second and third screening criteria for the key metabolic pathways described in Section Screening of the key metabolic pathways affected by GNPs and SNPs in Materials and methods (pathway involved candidate key metabolites and had the highest pathway impact), three pathways shown in Table 6 (lines 2, 3 and 5) were found to involve candidate key metabolites (glutathione and uridine) in the GNP group, and the ‘glutathione metabolism pathway’ had the highest impact and involved glutathione. Therefore, the ‘glutathione metabolism pathway’ was identified as the key pathway in the GNP group (Fig. 3). For the SNP group, all three pathways in Table 7 involved candidate key metabolites (malic acid), and the ‘citrate cycle pathway’ had the highest impact. Thus, the ‘citrate cycle pathway’ was the key pathway in the SNP group (Fig. 4).

In order to identify the key metabolites induced by GNPs and SNPs, molecular function analysis of candidate key metabolites involved in key metabolic pathways were further carried out in this article to determine their correlation with the effect of GNPs/SNPs on cells. Glutathione metabolism mainly involves the process of the synthesis of glutathione from glutamate. Glutathione is a linear tripeptide of glutamine, cysteine and glycine. It is the most important sulphydryl compound and the most abundant small molecule tripeptide compound in cells, and it is an important substance for the free radical defence system of the body. It can effectively remove free radicals produced by biological oxidation, maintain the stability of the intracellular environment and play an important role in protecting tissues exposed to the oxidative environment from free radical damage [1, 25, 26]. Glutathione is also involved in substance transport, the regulation of gene expression, DNA and protein synthesis, cell proliferation and apoptosis, cytokine production and immune responses [27, 28]. Glutathione comprises both reduced glutathione (GSH) and oxidized glutathione (GSSG). GSH is the main source of sulphydryl groups in most living cells plays an important role in maintaining the proper redox state of sulphydryl groups in proteins and is a key antioxidant in animal cells. Usually, 90–95% of total glutathione is GSH. GSSG can be reduced to GSH by glutathione reductase.

Glutathione can bind a variety of harmful chemicals that enter the body and their metabolites and may be a key substance for reducing the toxicity of these substances [26, 29–32]. Lu *et al.* found that glutathione had an antagonistic effect on the toxicity of levodopa. The combination of glutathione and levodopa could significantly reduce the levels of ROS and malondialdehyde in the substantia nigra of rats, enhance the activity of glutathione peroxidase and enhance the antioxidant capability of the body [25]. Sun *et al.* studied the protective effect of glutathione on arsenic-induced toxicity in HaCaT cells and found that glutathione supplementation *in vitro* can reduce the cytotoxicity of arsenic, indicating that glutathione has a significant detoxification effect on arsenic-induced cytotoxicity [32].

The chemical composition of GNPs includes the heavy metal gold. Some researchers believe that GNPs do not show cytotoxicity for two reasons: one is that they are not toxic to cells, and the other is that some substances in cells can reduce the toxicity of GNPs [33]. Li Wushan *et al.* showed that when glutathione in CHO-K1 cells was reduced by butyl sulphoxide-sulphoximine, 13 nm GNPs significantly reduced cell viability, changed the cell morphology, destroyed the microfilament structure and induced cell apoptosis. After exogenous glutathione was added, the cell survival rate and cell morphology of the GNP-treated group were restored, and the cells showed no obvious apoptosis, suggesting that glutathione is an important substance in cells that reduces GNP-induced toxicity [33]. In this paper, the increase in glutathione observed in GNP-treated HDFs after 4, 8 and 24 h (Table 3) might reflect a protective response in HDFs to reduce GNP-mediated oxidative damage and avoid the inhibition of cell proliferation (Fig. 2). So glutathione could be identified as the key metabolite induced by GNPs.

Oxidative stress induced by excessive ROS accumulation is considered the main mechanism by which SNPs exert toxicity [34]. Thus, the ability of cells to preserve glutathione-mediated defence mechanisms is critical for cellular redox homeostasis, and glutathione levels might be an indication of such an ability. Glutathione can protect cellular components from oxidative damage by directly neutralizing ROS or by acting as a cofactor for free radicals in cells [1]. In addition, as a thiol compound, glutathione can bind free metal ions with high affinity, reduce metal ion content, indirectly reduce ROS production and alleviate oxidative stress [35]. Several studies have reported that exposure to SNPs causes GSH depletion in different cell types (e.g. Caco-2 cells and keratinocytes) [20, 34, 36] and have proposed that this arises from the inhibition by GSH of enzyme synthesis or the increased conversion of GSH to the oxidized form (GSSG) [34]. The results in this study (Table 3) showed that glutathione in SNP-treated HDFs after 4 and 8 h was downregulated (fold change < 0), indicating that SNPs could cause the consumption of glutathione and reduce the resistance to SNP-induced oxidative stress, which in turn may have induced oxidative stress and ultimately resulted in cytotoxicity (Fig. 2).

The citrate cycle is the main way for the body to obtain energy. It is a common process used to completely oxidize sugar, fat and protein to release energy, and it is also a hub for their interaction and transformation. The citrate cycle is a step in the process of respiration, after which high-energy electrons undergo oxidative phosphorylation through the electron transport chain with the aid of NAHD+H^+^ and FADH2 to generate a large amount of energy.

Studies have shown that exposure to nanomaterials could affect the citrate cycle in the body. SNPs with a size of 30 nm reduced the activity of the citrate cycle in human keratinocytes after 48 h of treatment [1]. Treatment with 8 nm SNPs at 30 μg/ml in CeO_2_-treated HepG2 cells caused the downregulation of three genes in the TCA cycle [37].

Malic acid is an active substance in living cells and an important organic acid produced by the metabolism of organisms. It is an important intermediate in the citrate cycle and the glyoxylate cycle. It can increase the activity of malate dehydrogenase to increase the levels of intermediates in the citrate cycle rapidly and thereby increase the speed of the citrate cycle [38, 39]. Malic acid can enter the matrix through the α-ketoglutarate transporter on the mitochondrial inner membrane and regenerate oxaloacetate and NADH via the activity of matrix dehydrogenase. NADH enters the electronic respiratory chain to produce ATP, so malic acid and ATP generation are closely related [38, 39]. Previous research has shown that supplementation with l-malic acid can significantly increase ATP production [40].

By using a metabolomics technique, Sun *et al.* found that five metabolites (l-aspartic acid, l-malic acid, myoinositol, d-sorbitol and citric acid) in the human hepatocyte cell line LO_2_ were downregulated and that l-cysteine was upregulated after treatment of cells with 100 mg/l SNPs for 24 h. The addition of l-malic acid significantly mitigated the decrease in cell viability induced by the SNPs [22], which implied that l-malic acid played a critical role in the control and restoration of cellular function [41].

The results of this study (Table 3) showed that after 4, 8 and 24 h of SNP treatment, malic acid was downregulated (fold change < 0), and the fold change in the SNP-24h group was the lowest. The downregulated expression of malic acid could decrease the speed of the citrate cycle, which in turn could inhibit the production of ATP and cell viability. These results were consistent with the results that showed a significant decrease in cell proliferation after 24 h of SNP treatment (Fig. 2). Therefore, malic acid could be identified as the key metabolite induced by SNPs.

Citrate is an important intermediate in the citrate cycle that links glycolysis and the citrate cycle. It is of great importance for the metabolism of sugars and fatty acids. Citrate produces isocitrate during glucose metabolism, inhibiting the activity of phosphofructokinase and pyruvate dehydrogenase [42, 43]. Citrate can be transported from the mitochondria by the citrate-malate shuttle and then converted back into acetyl-CoA for fatty acid synthesis. As an upstream metabolite in the citrate cycle, citrate can affect the metabolism of malic acid, and excessive citrate can inhibit the downstream production of malic acid [44].

According to the above analysis, GNPs might induce the upregulation of glutathione in a key metabolic pathway (glutathione metabolic pathway), triggering the mechanism involved in protection from intracellular oxidative stress and ultimately reducing cytotoxicity. Meanwhile, SNPs might affect the level of citrate by regulating a key metabolic pathway (citrate cycle pathway), which in turn might downregulate a key metabolite (malic acid) to further inhibit ATP production and ultimately lead to cytotoxicity. Therefore, this study verified the function of the metabolic pathway in terms of three aspects: total glutathione content, ATP content and citrate content.

### Verification of the expression levels of key metabolites involved in key metabolic pathways and the pathway functions affected by GNPs and SNPs

#### Verification of the expression levels of key metabolites

In order to eliminate the false positive or false negative results caused by technical errors in high-throughput experiments and signal analysis, etc., the expression levels of key metabolites induced by GNPs and SNPs were further verified in this article.

**Figure 5:**
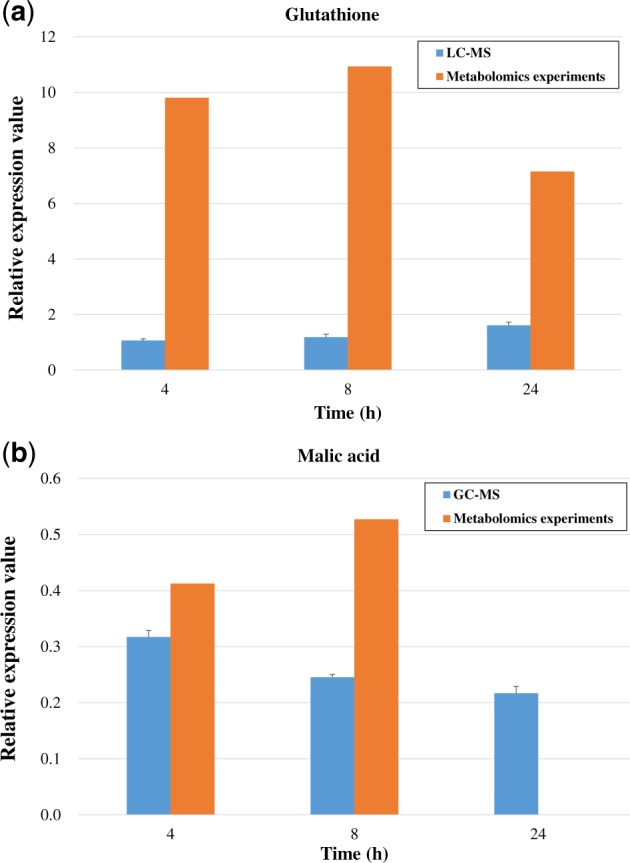
Validation of the expression of the two key metabolites involved in the key metabolic pathways in comparison with the metabolomics experimental results: (**a**) glutathione in the GNP group; (**b**) malic acid in the SNP group

The expression levels of glutathione (a key metabolite involved in the glutathione metabolic pathway in the GNP group) and malic acid (a key metabolite involved in the citrate cycle pathway in the SNP group) were detected by LC-MS and GC-MS, respectively. The results are shown in Fig. 5 and are compared with the results from the metabolomics experiments. The expression levels of the two key metabolites determined by the two tested methods were consistent: glutathione was upregulated in the GNP group (relative expression value >1), and malic acid was downregulated in the SNP group (relative expression value <1).

#### Verification of the functions of key metabolic pathways

Because the functions of the key metabolic pathways affected by the GNPs and SNPs were mainly related to glutathione synthesis and energy metabolism, the effects of GNPs on the total glutathione contents and the effect of SNPs on ATP and citrate production were analysed here.

##### Determination of the total glutathione content

**Figure 6:**
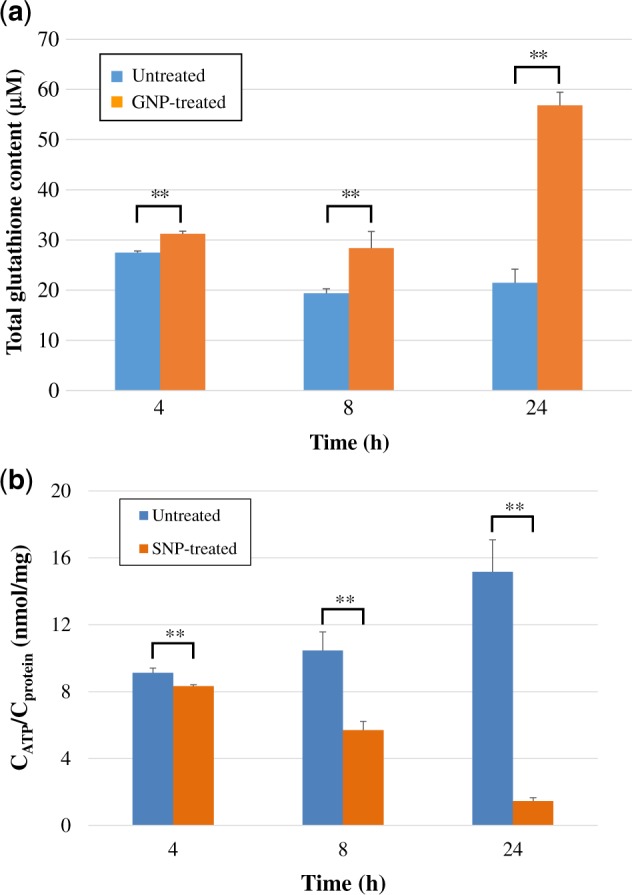
(**a**) The total glutathione content in untreated and 200 μM GNP-treated HDFs after 4, 8 and 24 h. **P <* *0.01 indicates a very significant difference compared to the untreated group. (**b**) The ATP content in untreated and 200 μM SNP-treated HDFs after 4, 8 and 24 h

The total glutathione content in untreated and GNP-treated HDFs is shown in Fig. 6a. The total glutathione content after GNP treatment was significantly higher than that in the control group (*P *<* *0.01), indicating that GNPs could lead to an increase in total glutathione content in HDFs. The results agreed with the results showing the upregulation of glutathione obtained from the metabolomics and validation experiments (Table 2 and Fig. 5a).

##### Measurement of ATP content

Cell growth requires glycolysis for ATP generation to maintain the energy supply and to accumulate glycolytic intermediates to meet the needs of rapid cell proliferation and the rapid synthesis of nucleotides, lipids and proteins [45]. The intracellular ATP contents in untreated and SNP-treated HDFs were measured and are shown in Fig. 6b. The ATP content in all SNP-treated HDFs was significantly lower than that in the untreated group (*P *<* *0.01), indicating that the SNPs could cause a large decrease in ATP content. The results were consistent with the downregulation of malic acid (Fig. 5b), which confirmed that a decrease in malic acid could inhibit the production of ATP. This was also consistent with the results reported in the previous literatures that showed that SNPs could lead to mitochondrial dysfunction and a decrease in the ATP content in cells [10, 46]. A decrease in ATP content could further affect cell proliferation (Fig. 2).

##### Detection of citrate content

**Figure 7:**
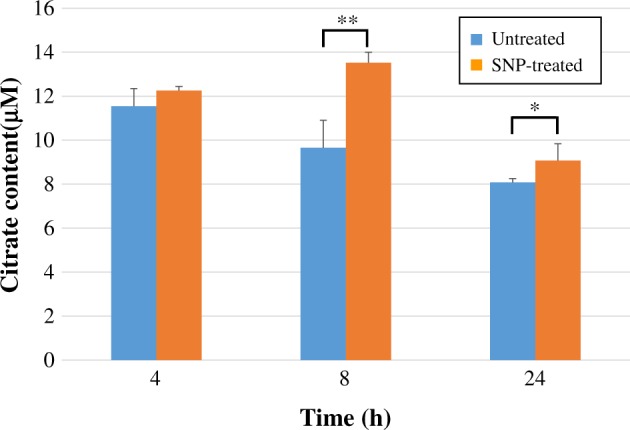
The citrate content in untreated and 200 μM SNP-treated HDFs after 4, 8 and 24 h

The citrate content in untreated and SNP-treated HDFs was detected and is shown in Fig. 7. The citrate content in SNP-treated cells was higher than that in the untreated groups. The citrate content in the SNP-8h group was significantly higher than that in the untreated group (*P *<* *0.01), and it was also significantly higher than that in the untreated group after 24 h of treatment (*P *<* *0.05).

Citrate is a product of energy metabolism, and its concentration will increase significantly when metabolism is not balanced. Huang *et al.* found that citrate could arrest CHO cells in G1 phase, hinder DNA synthesis and inhibit cell proliferation [47]. This study also showed that citrate content was increased after SNP treatment and might have had an effect on cell proliferation, which is consistent with the cytotoxicity results for the SNPs (Fig. 2). In addition, excess citrate inhibits the activity of citrate synthase. Citrate synthase is the rate-limiting enzyme in the citrate cycle. When it is inhibited, the condensation reaction between oxaloacetate and acetyl-CoA will occur more slowly, and the metabolism of the citrate cycle pathway will be attenuated, thereby inhibiting the downstream production of malic acid [42] and resulting in a decrease in malic acid content (Fig. 5b).

**Figure 8:**
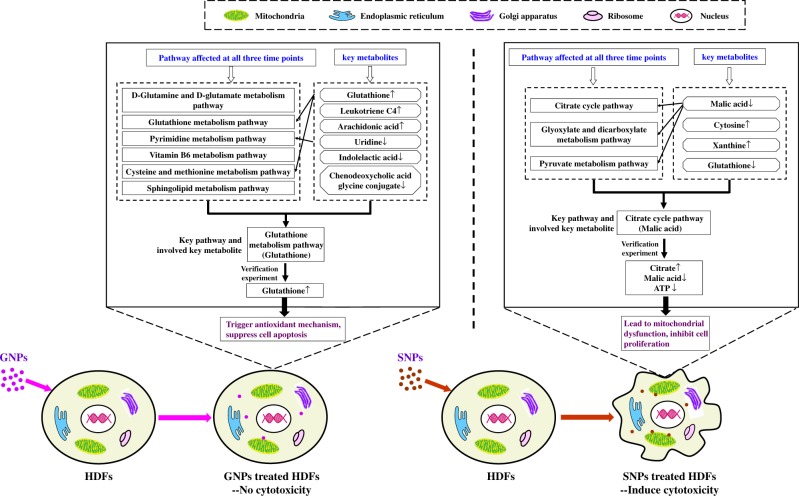
Comparison of the interactions between GNPs/SNPs and HDFs

So, based on above metabolomics analysis and verification experiments, it was found that the increase in glutathione content caused by GNPs might trigger a protective intracellular response to GNP-mediated oxidative stress and ultimately reduce cytotoxicity. SNP treatment increased the citrate content and decreased malic acid through the citrate cycle pathway, which further affected ATP production and ultimately led to cytotoxicity (Fig. 8).

## Conclusion

The analysis at the cellular level shown in this paper revealed that 200 μM GNPs and SNPs (20 nm) had different effects on cell proliferation. Metabolomic techniques were used to compare the metabolite expression profiles in HDFs after treatment with GNPs and SNPs for different amounts of time. By analysing the expression patterns of the differentially expressed metabolites and metabolic pathways, the key metabolites and key metabolic pathways involved in the interactions between GNPs/SNPs and HDFs were identified. Based on the verification experiments conducted on the key metabolites and key metabolic pathways, different effects of GNPs and SNPs with different cytotoxicities on HDFs at the metabolic level were illustrated.

## Supplementary Material

rbz051_Supplementary_Data
