# Supplementary material for: Comparative study of the effects of gold and silver nanoparticles on the metabolism of human dermal fibroblasts
Source: Regen Biomater. 2020 Jan 25;7(2):221–32. doi: 10.1093/rb/rbz051 (PMC7147366; doi:10.1093/rb/rbz051)
Supplement: rbz051_Supplementary_Data [file rbz051_supplementary_data.docx]

**Supporting Information**

**Title:** Comparative study of the effects of gold and silver nanoparticles on the metabolism of human dermal fibroblasts

**Author:** Yan Huang, Xiaoying Lü*, Rong Chen, Ye Chen

**Supplementary Table 1:** Diffenentially expressed metabolites in GNPs-4h group.

**Supplementary Table 2:** Diffenentially expressed metabolites in GNPs-8h group.

**Supplementary Table 3:** Diffenentially expressed metabolites in GNPs-24h group.

**Supplementary Table 4:** Diffenentially expressed metabolites in SNPs-4h group.

**Supplementary Table 5:** Diffenentially expressed metabolites in SNPs-8h group.

**Supplementary Table 6:** Diffenentially expressed metabolites in SNPs-24h group.

**Supplementary Table 7:** Metabolic pathways affected by the differentially expressed metabolites in the GNPs-4h group and the involved metabolites and pathway impact.

**Supplementary Table 8:** Metabolic pathways affected by the differentially expressed metabolites in the GNPs-8h group and the involved metabolites and pathway impact.

**Supplementary Table 9:** Metabolic pathways affected by the differentially expressed metabolites in the GNPs-24h group and the involved metabolites and pathway impact.

**Supplementary Table 10:** Metabolic pathways affected by the differentially expressed metabolites in the SNPs-4h group and the involved metabolites and pathway impact.

**Supplementary Table 11:** Metabolic pathways affected by the differentially expressed metabolites in the SNPs-8h group and the involved metabolites and pathway impact.

**Supplementary Table 12:** Metabolic pathways affected by the differentially expressed metabolites in the SNPs-24h group and the involved metabolites and pathway impact.

**Supplementary Table 1:** Diffenentially expressed metabolites in GNPs-4h group.

| No. | Name of metabolite | RT(min) | Mass | VIP value | *P* value | Fold change |
| --- | --- | --- | --- | --- | --- | --- |
| 1 | Glutathione | 1.12 | 307.0845 | 2.203 | 0.000 | 3.294 |
| 2 | Linoleamide | 13.05 | 279.257 | 1.575 | 0.040 | 1.948 |
| 3 | Leukotriene C4 | 11.496 | 625.3131 | 1.954 | 0.020 | 1.505 |
| 4 | Glutamate | 0.76 | 147.0531 | 2.204 | 0.000 | 1.213 |
| 5 | Threonine | 0.75 | 119.0583 | 1.687 | 0.024 | 0.949 |
| 6 | Arachidonic Acid | 13.602 | 304.2404 | 2.232 | 0.004 | 0.701 |
| 7 | PE(20:4) | 10.61 | 501.2868 | 1.787 | 0.014 | 0.580 |
| 8 | PE(22:4) | 11.597 | 529.3185 | 1.988 | 0.017 | 0.547 |
| 9 | PE(22:6) | 10.60 | 525.2876 | 1.860 | 0.009 | 0.512 |
| 10 | Docosahexaenoic acid | 13.440 | 328.2409 | 1.895 | 0.026 | 0.482 |
| 11 | Choline | 0.74 | 103.0999 | 1.673 | 0.025 | 0.399 |
| 12 | α-Tocopherol | 14.13 | 430.3779 | 1.561 | 0.042 | 0.353 |
| 13 | PC(13:0)/PE(16:0) | 10.91 | 453.2867 | 1.668 | 0.026 | 0.284 |
| 14 | Creatine | 0.75 | 131.0694 | 1.840 | 0.010 | 0.222 |
| 15 | Tetradecanedioic acid | 11.96 | 258.184 | 1.566 | 0.041 | 0.214 |
| 16 | Arginine | 0.70 | 174.1119 | 1.626 | 0.032 | 0.206 |
| 17 | Uridine | 1.127 | 244.0701 | 1.919 | 0.024 | -0.260 |
| 18 | Oxododecanoic acid | 10.52 | 214.1573 | 1.579 | 0.039 | -0.344 |
| 19 | Indolelactic acid | 5.987 | 205.074 | 1.805 | 0.038 | -0.361 |
| 20 | Chenodeoxycholic acid glycine conjugate | 9.425 | 449.3156 | 2.356 | 0.001 | -0.499 |
| 21 | DiHODE/HpODE | 10.727 | 312.2307 | 2.025 | 0.014 | -0.513 |
| 22 | Palmitic acid | 14.239 | 256.2401 | 1.808 | 0.037 | -0.638 |
| 23 | Phytosphingosine | 8.65 | 317.2942 | 1.593 | 0.037 | -0.881 |
| 24 | kamlolenic acid | 10.73 | 294.2205 | 1.552 | 0.044 | -1.197 |
| 25 | Decanoyl-L-carnitine | 8.14 | 315.2419 | 1.698 | 0.022 | -1.286 |
| 26 | Hydroxyphenyllactic acid | 4.062 | 182.0579 | 2.031 | 0.014 | -1.608 |
| 27 | PGA2 | 9.916 | 334.2151 | 1.777 | 0.042 | -2.377 |
| 28 | dehydrocholic acid | 12.49 | 402.2422 | 1.848 | 0.009 | -2.914 |
| 29 | Anandamide (20:2, n-6) | 13.17 | 351.314 | 2.333 | 0.000 | -3.057 |

**Supplementary Table 2:** Diffenentially expressed metabolites in GNPs-8h group.

| No. | Name of metabolite | RT(min) | Mass | VIP value | *P* value | Fold change |
| --- | --- | --- | --- | --- | --- | --- |
| 1 | PC(19:3) | 12.25 | 531.3338 | 2.143 | 0.011 | 3.776 |
| 2 | Glutathione | 1.12 | 307.0845 | 1.828 | 0.017 | 3.451 |
| 3 | Acetylneuraminic Acid | 0.79 | 309.1064 | 1.596 | 0.049 | 2.498 |
| 4 | α-Tocopherol | 14.07 | 430.3782 | 1.721 | 0.029 | 2.188 |
| 5 | Linoleamide | 13.05 | 279.257 | 2.113 | 0.002 | 2.134 |
| 6 | Xanthine | 1.13 | 152.0326 | 1.918 | 0.032 | 1.956 |
| 7 | Leukotriene C4 | 11.50 | 625.3131 | 1.826 | 0.044 | 1.386 |
| 8 | Glutamate | 0.76 | 147.0531 | 1.629 | 0.043 | 0.871 |
| 9 | PE(22:4) | 11.45 | 529.3181 | 2.152 | 0.002 | 0.618 |
| 10 | Arachidonic Acid | 13.60 | 304.2404 | 2.224 | 0.007 | 0.536 |
| 11 | PE(22:6) | 10.60 | 525.2876 | 1.829 | 0.017 | 0.445 |
| 12 | Docosahexaenoic acid | 13.44 | 328.2409 | 1.872 | 0.037 | 0.379 |
| 13 | PE(20:4) | 10.61 | 501.2868 | 1.758 | 0.025 | 0.322 |
| 14 | Palmitic amide | 13.37 | 255.2576 | 1.597 | 0.049 | -0.192 |
| 15 | Uridine | 1.13 | 244.0701 | 1.908 | 0.033 | -0.262 |
| 16 | Pyroglutamic acid | 0.84 | 129.0428 | 1.901 | 0.034 | -0.270 |
| 17 | Uric acid | 0.80 | 168.0286 | 2.211 | 0.008 | -0.306 |
| 18 | Glycocholic Acid | 8.20 | 465.3107 | 2.108 | 0.014 | -0.384 |
| 19 | Indolelactic acid | 5.99 | 205.074 | 2.164 | 0.010 | -0.413 |
| 20 | Chenodeoxycholic acid glycine conjugate | 9.42 | 449.3156 | 2.476 | 0.001 | -0.429 |
| 21 | HETE | 11.73 | 320.2358 | 1.859 | 0.039 | -0.456 |
| 22 | DiHODE/HpODE | 10.73 | 312.2307 | 2.256 | 0.006 | -0.639 |
| 23 | TriHOME | 9.40 | 330.2414 | 2.037 | 0.019 | -0.731 |
| 24 | Hippuric acid | 4.84 | 179.0582 | 1.804 | 0.048 | -0.790 |
| 25 | Dopaquinone | 6.49 | 195.0531 | 2.002 | 0.022 | -0.817 |
| 26 | Sphingosine | 9.82 | 299.2835 | 2.053 | 0.004 | -0.870 |
| 27 | Phytosphingosine | 8.65 | 317.2942 | 1.847 | 0.016 | -1.140 |
| 28 | oxo-dodecanoic acid | 10.95 | 214.1571 | 1.651 | 0.039 | -1.339 |
| 29 | methyl palmitic acid | 13.13 | 270.2563 | 1.618 | 0.045 | -1.809 |
| 30 | Decanoyl-L-carnitine | 8.14 | 315.2419 | 2.027 | 0.005 | -2.025 |

**Supplementary Table 3:** Diffenentially expressed metabolites in GNPs-24h group.

| No. | Name of metabolite | RT(min) | Mass | VIP value | *P* value | Fold change |
| --- | --- | --- | --- | --- | --- | --- |
| 1 | Cytosine | 1.13 | 111.0438 | 1.93 | 0.000 | 16.021 |
| 2 | Glutathione | 1.12 | 307.0845 | 1.81 | 0.001 | 2.839 |
| 3 | 5-Amino-6-(5'-phosphoribosylamino)uracil | 6.26 | 354.0574 | 1.873 | 0.005 | 2.306 |
| 4 | Sphingosine | 10.27 | 299.2834 | 1.90 | 0.000 | 2.132 |
| 5 | Sphinganine | 9.50 | 301.2995 | 2.02 | 0.000 | 2.131 |
| 6 | PE(22:6) | 10.60 | 525.2895 | 1.893 | 0.004 | 1.961 |
| 7 | C16 Sphinganine | 8.58 | 273.2681 | 2.00 | 0.000 | 1.632 |
| 8 | Phytosphingosine | 8.65 | 317.2942 | 1.98 | 0.000 | 1.503 |
| 9 | PC(15:1)/PE(18:1) | 11.45 | 479.3029 | 1.825 | 0.007 | 1.371 |
| 10 | LysoPE(22:5) | 10.86 | 527.303 | 1.623 | 0.024 | 1.015 |
| 11 | PE(20:4) | 10.77 | 501.2867 | 1.98 | 0.000 | 0.923 |
| 12 | PC(13:0)/PE(16:0) | 11.12 | 453.2868 | 2.07 | 0.000 | 0.839 |
| 13 | PE(22:4) | 11.60 | 529.3185 | 2.055 | 0.001 | 0.743 |
| 14 | Palmitoyl-L-carnitine | 10.76 | 399.3365 | 1.95 | 0.000 | 0.668 |
| 15 | Elaidic carnitine | 10.96 | 425.3518 | 1.81 | 0.001 | 0.589 |
| 16 | PC(15:0)/PE(18:0) | 12.32 | 481.3186 | 1.880 | 0.004 | 0.501 |
| 17 | Stearoylcarnitine | 11.49 | 427.3676 | 1.83 | 0.001 | 0.500 |
| 18 | Palmitic amide | 13.37 | 255.2576 | 1.75 | 0.003 | 0.313 |
| 19 | Oxododecanoic acid | 10.95 | 214.1571 | 1.35 | 0.044 | -0.196 |
| 20 | lauroleic acid | 10.39 | 198.1624 | 1.44 | 0.028 | -0.196 |
| 21 | Oleamide | 12.57 | 281.2729 | 1.34 | 0.045 | -0.247 |
| 22 | Creatine | 0.75 | 131.0694 | 1.76 | 0.002 | -0.294 |
| 23 | N-palmitoyl alanine | 12.57 | 327.2784 | 1.38 | 0.038 | -0.326 |
| 24 | α-Tocopherol | 14.13 | 430.3779 | 1.87 | 0.000 | -0.577 |
| 25 | Deoxyuridine monophosphate (dUMP) | 1.13 | 308.043 | 1.686 | 0.017 | -0.591 |
| 26 | Glutamate | 0.76 | 147.0535 | 1.517 | 0.040 | -1.060 |
| 27 | Malic acid | 0.93 | 134.0217 | 1.777 | 0.010 | -1.529 |

**Supplementary Table 4:** Diffenentially expressed metabolites in SNPs-4h group.

| No. | Name of metabolite | RT(min) | Mass | VIP value | *P* value | Fold change |
| --- | --- | --- | --- | --- | --- | --- |
| 1 | Glutathione | 1.12 | 307.0845 | 2.100 | 0.006 | 3.560 |
| 2 | Hydroxyvaleric acid | 3.62 | 118.063 | 2.503 | 0.006 | 2.894 |
| 3 | Xanthine | 1.13 | 152.0326 | 2.472 | 0.007 | 2.349 |
| 4 | α-Tocopherol | 14.07 | 430.3782 | 1.773 | 0.034 | 1.153 |
| 5 | Glutamate | 0.76 | 147.0531 | 1.990 | 0.012 | 0.856 |
| 6 | Arachidonic Acid | 13.60 | 304.2404 | 2.580 | 0.004 | 0.711 |
| 7 | Chenodeoxycholic acid glycine conjugate | 9.42 | 449.3156 | 2.034 | 0.044 | -0.300 |
| 8 | oxo-dodecanoic acid | 10.95 | 214.1571 | 1.824 | 0.028 | -0.339 |
| 9 | Phytosphingosine | 8.65 | 317.2942 | 1.786 | 0.032 | -0.893 |
| 10 | Sphingosine | 9.82 | 299.2835 | 2.279 | 0.001 | -1.224 |
| 11 | Malic acid | 0.93 | 134.0217 | 1.992 | 0.050 | -1.277 |
| 12 | Anandamide (20:2, n-6) | 13.17 | 351.314 | 2.508 | 0.000 | -1.439 |
| 13 | Pipecolic acid | 1.12 | 129.0789 | 2.077 | 0.007 | -17.570 |

**Supplementary Table 5:** Diffenentially expressed metabolites in SNPs-8h group.

| No. | Name of metabolite | RT(min) | Mass | VIP value | *P* value | Fold change |
| --- | --- | --- | --- | --- | --- | --- |
| 1 | Cytosine | 1.13 | 111.0438 | 2.045 | 0.000 | 16.078 |
| 2 | Glutathione | 1.12 | 307.0845 | 1.539 | 0.021 | 2.925 |
| 3 | Hydroxyvaleric acid | 3.62 | 118.063 | 1.933 | 0.015 | 2.916 |
| 4 | 5-Amino-6-(5'-phosphoribosylamino)uracil | 6.26 | 354.0574 | 2.257 | 0.001 | 1.933 |
| 5 | Xanthine | 1.13 | 152.0326 | 1.726 | 0.039 | 1.815 |
| 6 | PC(13:0)/PE(16:0) | 11.11 | 453.2872 | 2.030 | 0.009 | 1.675 |
| 7 | Sphingosine | 10.27 | 299.2834 | 1.834 | 0.002 | 1.644 |
| 8 | Leukotriene C4 | 11.50 | 625.3131 | 1.899 | 0.018 | 1.610 |
| 9 | Aminooctanoic acid | 0.80 | 159.1259 | 1.476 | 0.030 | 1.470 |
| 10 | Sphinganine | 9.50 | 301.2995 | 2.003 | 0.000 | 1.214 |
| 11 | C16 Sphinganine | 8.58 | 273.2681 | 1.929 | 0.001 | 1.066 |
| 12 | PC(20:4) | 10.66 | 543.3338 | 1.414 | 0.040 | 0.761 |
| 13 | Pyroglutamic acid | 1.13 | 129.0428 | 1.580 | 0.016 | 0.627 |
| 14 | Elaidic carnitine | 10.96 | 425.3518 | 1.865 | 0.001 | 0.504 |
| 15 | Valine | 1.12 | 117.0792 | 1.429 | 0.038 | 0.475 |
| 16 | Phenylpyruvic acid | 1.18 | 164.0477 | 1.393 | 0.044 | 0.450 |
| 17 | Phenylalanine | 2.19 | 165.0793 | 1.471 | 0.030 | 0.445 |
| 18 | PE(20:4) | 10.62 | 501.2873 | 1.745 | 0.036 | 0.386 |
| 19 | Palmitoyl-L-carnitine | 10.76 | 399.3365 | 1.736 | 0.005 | 0.354 |
| 20 | PC(18:2) | 10.60 | 519.3336 | 1.535 | 0.021 | 0.346 |
| 21 | Stearoylcarnitine | 11.49 | 427.3676 | 1.671 | 0.009 | 0.324 |
| 22 | L-Isoleucine/ L-Leucine | 0.85 | 131.0946 | 1.658 | 0.010 | 0.319 |
| 23 | Arginine | 0.70 | 174.1119 | 1.483 | 0.029 | 0.247 |
| 24 | Dopaquinone | 6.49 | 195.0531 | 1.705 | 0.042 | 0.210 |
| 25 | lauroleic acid | 10.39 | 198.1624 | 1.496 | 0.027 | -0.418 |
| 26 | Glucose | 0.74 | 180.064 | 1.838 | 0.024 | -0.473 |
| 27 | Uridine | 0.80 | 244.0703 | 1.913 | 0.017 | -0.535 |
| 28 | dodecanamide | 10.79 | 199.1941 | 1.529 | 0.022 | -0.569 |
| 29 | Palmitic acid | 14.24 | 256.2401 | 1.750 | 0.035 | -0.640 |
| 30 | Deoxyuridine monophosphate (dUMP) | 1.13 | 308.043 | 1.715 | 0.040 | -0.794 |
| 31 | Malic acid | 0.93 | 134.0217 | 1.678 | 0.046 | -0.923 |
| 32 | oxo-dodecanoic acid | 10.95 | 214.1571 | 1.496 | 0.027 | -1.111 |
| 33 | PC(19:3) | 12.22 | 531.3336 | 1.448 | 0.034 | -1.757 |

**Supplementary Table 6:** Diffenentially expressed metabolites in SNPs-24h group.

| No. | Name of metabolite | RT(min) | Mass | VIP value | *P* value | Fold change |
| --- | --- | --- | --- | --- | --- | --- |
| 1 | Cytosine | 1.13 | 111.0438 | 2.271 | 0.009 | 14.383 |
| 2 | dodecadienoic acid | 9.899 | 196.1464 | 1.543 | 0.043 | 2.254 |
| 3 | tridecadienoic acid | 8.69 | 210.1626 | 1.920 | 0.041 | 0.955 |
| 4 | Glucose | 0.737 | 180.064 | 1.778 | 0.013 | 0.660 |
| 5 | Oleic Acid | 14.404 | 282.256 | 1.680 | 0.023 | 0.473 |
| 6 | Choline | 0.74 | 103.0999 | 1.876 | 0.048 | -1.362 |
| 7 | α-Tocopherol | 14.13 | 430.3779 | 2.244 | 0.010 | -2.583 |
| 8 | Anandamide (20:2, n-6) | 13.17 | 351.314 | 1.939 | 0.039 | -2.645 |
| 9 | Glucoheptonic acid | 0.734 | 226.0697 | 1.682 | 0.023 | -3.093 |
| 10 | Palmitoyl-L-carnitine | 10.76 | 399.3365 | 2.084 | 0.022 | -3.291 |
| 11 | PE(22:4) | 11.45 | 529.3181 | 2.607 | 0.001 | -3.348 |
| 12 | Stearoylcarnitine | 11.49 | 427.3676 | 2.361 | 0.005 | -3.386 |
| 13 | PE(20:4) | 10.61 | 501.2868 | 2.568 | 0.001 | -3.581 |
| 14 | Palmitic amide | 13.37 | 255.2576 | 2.335 | 0.006 | -3.742 |
| 15 | Stearamide | 14.44 | 283.2886 | 1.991 | 0.032 | -4.124 |
| 16 | PC(13:0)/PE(16:0) | 10.91 | 453.2867 | 2.920 | 0.000 | -14.857 |
| 17 | PE(20:3) | 11.328 | 503.3026 | 1.931 | 0.005 | -17.376 |
| 18 | Malic acid | 0.926 | 134.0217 | 2.112 | 0.001 | -19.181 |
| 19 | PC(19:3) | 12.140 | 531.3339 | 2.242 | 0.000 | -19.755 |
| 20 | LysoPE(22:5) | 10.860 | 527.303 | 1.911 | 0.005 | -19.902 |
| 21 | PC(15:1)/PE(18:1) | 11.448 | 479.3029 | 1.542 | 0.044 | -22.541 |
| 22 | PC(15:0)/PE(18:0) | 12.324 | 481.3186 | 2.315 | 0.000 | -23.247 |

**Supplementary Table 7:** Metabolic pathways affected by the differentially expressed metabolites in the GNPs-4h group and the involved metabolites and pathway impact.

| No. | Metabolic pathway | Involved metabolite | Pathway impact |
| --- | --- | --- | --- |
| 1 | D-Glutamine and D-glutamate metabolism | D-Glutamic acid | 0.3262 |
| 2 | Arachidonic acid metabolism | Arachidonic acid  Leukotriene C4 | 0.25007 |
| 3 | Glutathione metabolism | Glutathione | 0.23743 |
| 4 | Arginine and proline metabolism | L-Arginine  Creatine | 0.15795 |
| 5 | Glycine, serine and threonine metabolism | Choline  L-Threonine  Creatine | 0.09732 |
| 6 | Ubiquinone and other terpenoid-quinone biosynthesis | 3-(4-Hydroxyphenyl)lactate | 0.0368 |
| 7 | Fatty acid metabolism | Palmitic acid | 0.02959 |
| 8 | Glycerophospholipid metabolism | Choline | 0.0212 |
| 9 | Pyrimidine metabolism | Uridine | 0.02061 |
| 10 | Primary bile acid biosynthesis | Chenodeoxycholic acid glycine conjugate | 0.00992 |
| 11 | Vitamin B6 metabolism | D-Glutamic acid | 0.00798 |
| 12 | Cysteine and methionine metabolism | Glutathione | 0.00735 |
| 13 | Sphingolipid metabolism | PhytoSphingosine | 0 |
| 14 | Tyrosine metabolism | 3-(4-Hydroxyphenyl)lactate | 0 |
| 15 | Fatty acid biosynthesis | Palmitic acid | 0 |
| 16 | Fatty acid elongation in mitochondria | Palmitic acid | 0 |
| 17 | Aminoacyl-tRNA biosynthesis | L-Arginine  L-Threonine | 0 |
| 18 | D-Arginine and D-ornithine metabolism | L-Arginine | 0 |
| 19 | Valine, leucine and isoleucine biosynthesis | L-Threonine | 0 |
| 20 | Tryptophan metabolism | Indolelactic acid | 0 |
| 21 | Porphyrin and chlorophyll metabolism | L-Threonine | 0 |

**Supplementary Table 8:** Metabolic pathways affected by the differentially expressed metabolites in the GNPs-8h group and the involved metabolites and pathway impact.

| No. | Metabolic pathway | Involved metabolite | Pathway impact |
| --- | --- | --- | --- |
| 1 | D-Glutamine and D-glutamate metabolism | D-Glutamic acid | 0.3262 |
| 2 | Arachidonic acid metabolism | Arachidonic acid  Leukotriene C4  Prostaglandin I2 | 0.26738 |
| 3 | Glutathione metabolism | Glutathione  Pyroglutamic acid | 0.23933 |
| 4 | Sphingolipid metabolism | Sphingosine  PhytoSphingosine | 0.09061 |
| 5 | Purine metabolism | Xanthine  Uric acid | 0.04586 |
| 6 | Tyrosine metabolism | Dopaquinone | 0.03741 |
| 7 | Phenylalanine metabolism | HippUric acid | 0.0315 |
| 8 | Caffeine metabolism | Xanthine | 0.0305 |
| 9 | Pyrimidine metabolism | Uridine | 0.02061 |
| 10 | Primary bile acid biosynthesis | Chenodeoxycholic acid glycine conjugate  Glycocholic acid | 0.01838 |
| 11 | Vitamin B6 metabolism | D-Glutamic acid | 0.00798 |
| 12 | Cysteine and methionine metabolism | Glutathione | 0.00735 |
| 13 | Tryptophan metabolism | Indolelactic acid | 0 |

**Supplementary Table 9:** Metabolic pathways affected by the differentially expressed metabolites in the GNPs-24h group and the involved metabolites and pathway impact.

| No. | Metabolic pathway | Involved metabolite | Pathway impact |
| --- | --- | --- | --- |
| 1 | D-Glutamine and D-glutamate metabolism | D-Glutamic acid | 0.3262 |
| 2 | Glutathione metabolism | Glutathione | 0.23743 |
| 3 | Sphingolipid metabolism | Sphinganine  PhytoSphingosine  Sphingosine | 0.23081 |
| 4 | Riboflavin metabolism | 5-Amino-6-(5'-phosphoribosylamino)uracil | 0.07634 |
| 5 | Citrate cycle (TCA cycle) | Malic acid | 0.04361 |
| 6 | Arginine and proline metabolism | Creatine | 0.03163 |
| 7 | Glyoxylate and dicarboxylate metabolism | Malic acid | 0.0242 |
| 8 | Pyrimidine metabolism | Cytosine | 0.02127 |
| 9 | Vitamin B6 metabolism | D-Glutamic acid | 0.00798 |
| 10 | Cysteine and methionine metabolism | Glutathione | 0.00735 |
| 11 | Purine metabolism | Deoxyinosine | 0.00158 |
| 12 | Glycine, serine and threonine metabolism | Creatine | 0.00071 |
| 13 | Fatty acid metabolism | L-Palmitoylcarnitine | 0 |
| 14 | Pyruvate metabolism | Malic acid | 0 |

**Supplementary Table 10:** Metabolic pathways affected by the differentially expressed metabolites in the SNPs-4h group and the involved metabolites and pathway impact.

| No. | Metabolic pathway | Involved metabolite | Pathway impact |
| --- | --- | --- | --- |
| 1 | D-Glutamine and D-glutamate metabolism | D-Glutamic acid | 0.3262 |
| 2 | Glutathione metabolism | Glutathione | 0.23743 |
| 3 | Arachidonic acid metabolism | Arachidonic acid | 0.21669 |
| 4 | Sphingolipid metabolism | Sphingosine  PhytoSphingosine | 0.09061 |
| 5 | Citrate cycle (TCA cycle) | Malic acid | 0.04361 |
| 6 | Purine metabolism | Xanthine | 0.03617 |
| 7 | Caffeine metabolism | Xanthine | 0.0305 |
| 8 | Glyoxylate and dicarboxylate metabolism | Malic acid | 0.0242 |
| 9 | Primary bile acid biosynthesis | Chenodeoxycholic acid glycine conjugate | 0.00992 |
| 10 | Vitamin B6 metabolism | D-Glutamic acid | 0.00798 |
| 11 | Cysteine and methionine metabolism） | Glutathione | 0.00735 |
| 12 | Lysine degradation | Pipecolic acid | 5.00E-04 |
| 13 | Pyruvate metabolism | Malic acid | 0 |

**Supplementary Table 11:** Metabolic pathways affected by the differentially expressed metabolites in the SNPs-8h group and the involved metabolites and pathway impact.

| No. | Metabolic pathway | Involved metabolite | Pathway impact |
| --- | --- | --- | --- |
| 1 | Glutathione metabolism | Glutathione  Pyroglutamic acid | 0.23933 |
| 2 | Sphingolipid metabolism | Sphinganine  Sphingosine | 0.23081 |
| 3 | Phenylalanine metabolism | L-Phenylalanine  Phenylpyruvic acid | 0.16781 |
| 4 | Arginine and proline metabolism | L-Arginine | 0.12632 |
| 5 | Riboflavin metabolism | 5-Amino-6-(5'-phosphoribosylamino)uracil | 0.07634 |
| 6 | Citrate cycle (TCA cycle) | Malic acid | 0.04361 |
| 7 | Pyrimidine metabolism | Cytosine  Uridine | 0.04188 |
| 8 | Purine metabolism | Deoxyinosine  Xanthine | 0.03775 |
| 9 | Tyrosine metabolism | Dopaquinone | 0.03741 |
| 10 | Arachidonic acid metabolism | Leukotriene C4 | 0.03338 |
| 11 | Caffeine metabolism | Xanthine | 0.0305 |
| 12 | Fatty acid metabolism | Palmitic acid  L-Palmitoylcarnitine | 0.02959 |
| 13 | Glyoxylate and dicarboxylate metabolism | Malic acid | 0.0242 |
| 14 | Starch and sucrose metabolism | D-Glucose | 0.01703 |
| 15 | Valine, leucine and isoleucine biosynthesis | L-Valine | 0.01325 |
| 16 | Cysteine and methionine metabolism | Glutathione | 0.00735 |
| 17 | Galactose metabolism | D-Glucose | 0.00276 |
| 18 | Phenylalanine, tyrosine and tryptophan biosynthesis | Phenylpyruvic acid  L-Phenylalanine | 0.00062 |
| 19 | Fatty acid biosynthesis | Palmitic acid | 0 |
| 20 | Fatty acid elongation in mitochondria | Palmitic acid | 0 |
| 21 | Nitrogen metabolism | L-Phenylalanine | 0 |
| 22 | Aminoacyl-tRNA biosynthesis | L-Phenylalanine  L-Arginine  L-Valine | 0 |
| 23 | Pentose phosphate pathway | D-Glucose | 0 |
| 24 | Glycolysis or Gluconeogenesis | D-Glucose | 0 |
| 25 | Pyruvate metabolism | Malic acid | 0 |
| 26 | Amino sugar and nucleotide sugar metabolism | D-Glucose  Fructose 6-phosphate | 0 |
| 27 | D-Arginine and D-ornithine metabolism | L-Arginine | 0 |
| 28 | Pantothenate and CoA biosynthesis | L-Valine | 0 |
| 29 | Valine, leucine and isoleucine degradation | L-Valine | 0 |
| 30 | Methane metabolism | Fructose 6-phosphate | 0 |
| 31 | Propanoate metabolism | L-Valine | 0 |
| 32 | Pentose and glucuronate interconversions | Fructose 6-phosphate | 0 |

**Supplementary Table 12:** Metabolic pathways affected by the differentially expressed metabolites in the SNPs-24h group and the involved metabolites and pathway impact.

| No. | Metabolic pathway | Involved metabolite | Pathway impact |
| --- | --- | --- | --- |
| 1 | Citrate cycle (TCA cycle) | Malic acid | 0.04361 |
| 2 | Glyoxylate and dicarboxylate metabolism | Malic acid | 0.0242 |
| 3 | Pyrimidine metabolism | Cytosine | 0.02127 |
| 4 | Glycerophospholipid metabolism | Choline | 0.0212 |
| 5 | Starch and sucrose metabolism | D-Glucose | 0.01703 |
| 6 | Galactose metabolism | D-Glucose | 0.00276 |
| 7 | Glycine, serine and threonine metabolism | Choline | 0 |
| 8 | Fatty acid metabolism | L-Palmitoylcarnitine | 0 |
| 9 | Fatty acid biosynthesis | Oleic acid | 0 |
| 10 | Pentose phosphate pathway | D-Glucose | 0 |
| 11 | Glycolysis or Gluconeogenesis | D-Glucose | 0 |
| 12 | Pyruvate metabolism | Malic acid | 0 |
| 13 | Amino sugar and nucleotide sugar metabolism | D-Glucose | 0 |
